# Analyzing Predictors of Interviews Across Surgical and Nonsurgical Specialties in the Context of the Step 1 Pass/Fail Change: A Retrospective Study

**DOI:** 10.7759/cureus.101178

**Published:** 2026-01-09

**Authors:** Layla Ali, Samuel Salib, Brian Kwan, Adam Y Ali, Iyawnna Hazzard, Suhyoung Ahn, Orr Amar, Shreya Guha, Jose Puglisi, Angela Mihalic, Michael S Wong

**Affiliations:** 1 College of Medicine, California Northstate University College of Medicine, Elk Grove, USA; 2 Otolaryngology - Head and Neck Surgery, California Northstate University College of Medicine, Elk Grove, USA; 3 Surgery, California Northstate University College of Medicine, Elk Grove, USA; 4 Research, California Northstate University College of Medicine, Elk Grove, USA; 5 Biostatistics, California Northstate University College of Medicine, Elk Grove, USA; 6 Student Affairs, University of Texas Southwestern Medical Center, Dallas, USA; 7 Surgery/Plastic Surgery, California Northstate University College of Medicine, Elk Grove, USA

**Keywords:** clerkship honors, interviews, medical residency, residency match, texas star database

## Abstract

This study examines how the transition of the United States Medical Licensing Examination (USMLE) Step 1 to pass/fail (P/F) scoring has affected residency interview predictors across surgical and non-surgical specialties. Using Texas Seeking Transparency in Application to Residency (STAR) data from 2018 to 2024, we evaluated trends in Step 2 Clinical Knowledge (CK) scores, clerkship honors, research output, and extracurricular factors. Step 2 CK scores were consistently predictive of interviews across all specialties. Clerkship honors were the strongest predictors for most fields, especially surgical specialties. Research publications had significant predictive value in neurological surgery, orthopedic surgery, otolaryngology, and urology. A logistic regression model for surgical match success identified the number of interviews, clerkship honors, and Step 2 CK scores as significant predictors, with interviews showing the strongest effect.

These findings suggest that academic metrics remain central to residency selection and highlight the need for advising strategies that reflect post-Step 1 scoring changes.

## Introduction

Surgical specialties have been considered more competitive than non-surgical specialties, as correlated with the highest unmatched percentage. According to the most recent National Resident Matching Program (NRMP) results and data from 2024, surgical specialties continue to have the highest unmatched rates. The highest unmatched medical doctorate (MD) and doctor of osteopathy (DO) specialties include neurological surgery (27.1% MD and 70% DO), orthopedic surgery (23.1% MD and 37.6% DO), plastic surgery (22% MD and 50% DO), otolaryngology (16.2% MD and 16.7% DO), and vascular surgery (4% MD and 50% DO) [1]. As of January 2022, the United States Medical Licensing Examination (USMLE) Step 1 exam became pass/fail (P/F), instead of using the traditional numerical score. This designated the class of 2024 as the first class to apply for residency with a non-numerical score. 

For context, the USMLE Step 1 exam is a standardized test assessing medical students’ foundational science knowledge, and has historically served as a major screening tool for residency interviews. In a previous study surveying 30 otolaryngology residency programs, letters of recommendation, USMLE Step 1 score, and the mean number of abstracts, presentations, and publications were the top three most important factors that program directors evaluated for prospective candidates [2]. However, with USMLE Step 1 now becoming P/F, these same program directors predicted a shift in ranking criteria, with competitive specialties, letters of recommendation, number of research publications, and the number of research experiences (posters, oral presentations, abstracts, etc.) as the top three most important factors [2]. Unfortunately, this study had a low survey response rate of 40%; therefore, the low sample size is not sufficient to reliably predict trends in competitive specialty match criteria. In another study analyzing applicant metrics in those who applied for neurological surgery, researchers observed a statistically significant increase in the average number of research, work, and volunteer experiences (p < 0.001), and an increase in the number of applicants who had an additional degree (p = 0.007) since the USMLE Step 1 exam became P/F [3]. 

To our knowledge, there is no current literature statistically evaluating the applicant metrics of those who have successfully matched in all competitive surgical specialties since the USMLE Step 1 became P/F. Considering the aforementioned, we aim to assess the demographic, curricular, and non-curricular factors that impact the competitive dynamics of the match rate in competitive surgical specialties, compared to other non-competitive, non-surgical specialties, from 2018 to 2024, for students to use this study as guidance and insight into the upcoming residency application cycle. 

Therefore, we analyzed a wide array of applicant metrics that residency programs commonly consider during the selection process. These were categorized into two major domains: curricular and non-curricular factors. Curricular factors encompassed quantitative measures, such as USMLE Step 2 scores and the number of clerkship honors received. The USMLE Step 2 exam continues to be numerically scored and serves as a key standardized assessment of a medical student’s clinical judgment. Step 2 maintains a three-digit score format, typically ranging from 1 to 300. Scores also provide a percentile rank, indicating how the examinee compares to their peers. The minimum passing score, as of July 1, 2022, is 214 [4]. This numeric score provides residency program directors with a quantifiable and comparative measure of applicants’ clinical readiness. As Step 1 no longer offers a numerical score, Step 2 has gained increased importance in residency selection, particularly for competitive specialties. Higher Step 2 scores are now frequently used as an indicator of academic strength and have become a distinguishing factor in the interview selection process. Non-curricular metrics included research productivity (abstracts, presentations, and publications), leadership roles, volunteer experiences, and the number of research experiences. These metrics were chosen based on their historical and projected relevance to residency selection criteria, particularly considering the transition to the new Step 1 scoring system. By evaluating these variables across multiple specialties and comparing trends before and after the implementation of the Step 1 P/F policy, this study provides a view into how the match process is evolving, especially in fields of varied competitiveness. 

The primary objective of this study was to identify curricular and non-curricular predictors of residency interview invitations across selected surgical and non-surgical specialties, using Texas Seeking Transparency in Application to Residency (STAR) data from 2018 to 2024. Secondary objectives included (1) comparing application metrics before and after the USMLE Step 1 transition to P/F, and (2) evaluating the relative predictive value of academic performance, research productivity, leadership, and volunteer experiences on interview and match outcomes.

The preliminary findings of this study were presented as a poster at the AAO-HNSF National Meeting in Indianapolis, IN, from October 10 to 13, 2025.

## Materials and methods

This retrospective database study was conducted to analyze various surgical and nonsurgical residency matching outcomes, using data from the Texas STAR program. The study included all residency applicants from medical schools participating in the Texas STAR program, who applied to neurological surgery, general surgery, neurology, orthopedic surgery, otolaryngology, pathology, pediatrics, and urology residency programs between 2018 and 2024. These specialties were chosen due to having sufficient applicant responses for data analysis, and to ensure proper representation of three groups: classically competitive surgical specialties, moderately competitive surgical specialties, and foundational nonsurgical specialties. Given the voluntary-response nature of the Texas STAR program, and its variable response rates across institutions, we cross-validated findings with publicly available data from the NRMP. NRMP data were used to contextualize overall match rates and specialty competitiveness trends, thereby providing an external reference point and mitigating potential sampling bias inherent to the Texas STAR dataset. Institutional Review Board (IRB) exemption was obtained under protocol number 2408-02-169, as the study utilized de-identified data and posed minimal risk to participants.

Data sources

The Texas STAR database is a voluntary, national, multi-institutional program, designed to provide transparency in the residency application process. Data included self-reported residency application metrics, such as USMLE scores, number of programs applied to, interview invitations, and match outcomes. Applicants to each specialty were identified from the overall dataset using the specialty of interest reported in the database. For 2023, the Texas STAR had responses from 146 medical schools (34% response rate), and in 2024, 155 medical schools participated (26% response rate) [5].

NRMP’s Charting Outcomes in the Match reports were utilized to compare national, specialty-specific match outcomes, serving as a benchmark to ensure the reliability and representativeness of findings derived from Texas STAR.

Study population

Our study population consisted of medical students who applied to various residency programs and completed surveys within the Texas STAR database between 2018 and 2024. Texas STAR is a voluntary, multi-institutional program that collects self-reported residency application data from medical schools across the United States. Inclusion criteria for this analysis required applicants who reported demographic information, USMLE Step 2 scores, and metrics related to research productivity (abstracts, posters, presentations, publications, and research experiences), leadership, volunteer activities, and clerkship honors. Data from incomplete or missing survey responses were excluded from the final analysis. Included specialties were general surgery, neurological surgery, neurology, orthopedic surgery, otolaryngology, pathology, pediatrics, dermatology, and urology. Additionally, it is important to note that the 2024 match cycle was the first in which applicants applied with Step 1 being P/F.

To address limitations in response rates and ensure external validity, results from Texas STAR were contextualized with publicly available NRMP data. NRMP data provided nationally representative benchmarks on specialty-specific match rates and applicant competitiveness, allowing for comparison and validation of trends observed in the Texas STAR cohort.

Included specialties were general surgery, neurological surgery, orthopedic surgery, otolaryngology, and dermatology. Additionally, it is important to note that the 2024 match cycle was the first in which applicants applied with Step 1 reported only as P/F.

Data collection

Following IRB approval, a proposal outlining the study aims, methodologies, and expected outcomes was submitted, with contributions from a study advisor. Texas STAR personnel were responsible for data extraction and provided the necessary datasets for analysis. The data were reviewed to ensure completeness and accuracy prior to statistical analysis. De-identified information was used to maintain confidentiality, in accordance with data-sharing agreements.

All available NRMP Charting Outcomes in the Match reports, released publicly every two years, were collected from 2008 to 2024. Metrics underlying the match statistics for U.S. M.D. seniors in otolaryngology, plastic surgery, neurosurgery, dermatology, orthopedic surgery, and general surgery were recorded. Fields were thoroughly reviewed to ensure accuracy prior to statistical analysis.

Statistical analysis

Statistical analysis was conducted using R (R Project for Statistical Computing, Vienna, Austria, version 4.3.3). Descriptive statistics, including the mean, standard deviation, and frequencies, were calculated to summarize the demographic characteristics and application profiles of applicants to various surgical and non-surgical specialties. The Mann-Whitney U test was used to compare continuous and discrete numerical variables across years, as the data were not normally distributed and consisted of independent samples. Additionally, a logistic regression model was built to predict the number of interviews received per applicant in each specialty using various continuous and discrete numerical variables. Research items were defined as the total number of abstracts, posters, and oral presentations. To simplify data analysis, grouped data, including Step 2 scores and age, were averaged, such that the age group “25-27” was assigned a discrete numerical value of 26 to allow for further analysis.

Further exploratory analysis was performed to assess trends in match rates over time and to identify factors associated with successful match outcomes. These factors included, but were not limited to, applicant qualifications (e.g., USMLE Step 1 and Step 2 scores, number of interview invites, and research productivity) and the number of applications submitted.

A multiple linear regression model was built to predict the number of interviews based on multiple continuous and categorical factors. Additionally, a logistic regression model was built, with iterations focusing only on significant factors; the model was validated using goodness-of-fit tests and receiver operating characteristic (ROC) curves.

Ethical considerations

All data were de-identified prior to analysis, and an IRB exemption ensured compliance with ethical guidelines for retrospective studies. The results of the analysis were used solely for research purposes, to better understand the residency application process and to identify potential areas for improvement in the surgical specialty match process.

This methodology ensures a comprehensive and rigorous approach to analyzing the residency matching outcomes of multiple surgical and non-surgical specialty applicants in the Texas STAR database from 2018 to 2024.

## Results

Upon compilation of all Texas STAR data, the following sample sizes for each specialty were obtained, as shown below.

The total surveyed across all specialties was 12,866 unique applicants. After filtering applicants with missing data points and excluding those who did not match, we ultimately analyzed 10,527 applicants. The breakdown of this can be found in Table 1.

**Table 1:** Breakdown of Each Specialty Analyzed Source: [4]

Specialty	Number of Applicants Analyzed	N% of Total Matched Applicants
General Surgery	2,015	18.60%
Neurological Surgery	328	19.80%
Neurology	1,019	20.50%
Orthopedic Surgery	1,420	22.80%
Otolaryngology	659	26.80%
Pathology	459	10.90%
Pediatrics	4,034	20.10%
Urology	593	24.00%

An additional statistical summary is included in Table 2, showing the summary statistics for the average of the years between 2018 and 2024 for selected metrics. Such metrics include USMLE Step 2 scores, number of applications submitted, number of interviews, percentage of applicants with research years, number of publications, and number of clerkship honors.

**Table 2:** Summary of Selected Metrics Across 2018-2024 for All Included Specialties Demonstrates the change in the average number of applications based on specialty.

Specialty	Step 2 Mean (SD)	Applications Submitted Mean (SD)	Interviews Received Mean (SD)	% of Applicants Who Took Research Year (SD)	Publications Mean (SD)	Clerkship Honors Mean (SD)
Neurological Surgery	254.97 (10.25)	70.67 (29.15)	23.56 (11.70)	25.30 (43.54)	7.07 (4.17)	4.39 (2.51)
Neurology	248.69 (13.65)	31.57 (17.13)	16.74 (7.53)	12.17 (32.71)	2.66 (3.04)	3.13 (2.38)
Orthopedic Surgery	256.96 (9.66)	71.11 (41.03)	14.11 (8.47)	13.59 (34.28)	4.59 (3.97)	4.35 (2.36)
Otolaryngology	256.97 (9.41)	69.26 (30.50)	16.06 (8.63)	19.42 (39.59)	5.20 (3.73)	4.34 (2.35)
Pathology	244.06 (14.93)	23.84 (12.07)	16.15 (8.21)	19.39 (39.58)	2.59 (3.30)	2.35 (2.35)
Pediatrics	246.54 (13.83)	28.87 (14.80)	17.22 (7.42)	5.50 (22.81)	1.56 (2.31)	2.96 (2.38)
Surgery	251.12 (11.69)	55.47 (31.35)	16.66 (8.86)	5.86 (23.49)	2.60 (2.97)	3.41 (2.44)
Urology	253.15 (10.60)	67.08 (31.09)	18.82 (9.09)	10.79 (31.05)	4.19 (3.45)	4.02 (2.49)

Changes in application metrics between 2023 (last scored Step 1 applicants) and 2024 (first Step 1 P/F applicants)

Table 3 demonstrates the change in the average number of applications based on specialty. Statistically significant decreases were seen in neurological surgery, orthopedic surgery, otolaryngology, urology, and dermatology.

**Table 3:** Average Number of Applications Based on Specialty Between 2023 and 2024

Specialty	2023	2024	Mann-Whitney U Test
				Applications Submitted, Mean (SD)	Applications Submitted, Mean (SD)	Applications Submitted, p-value
	General Surgery	60.57 (37.01)	59.44 (35.70)	0.80
Neurological Surgery	73.04 ± 28.25	50.32 (24.71)	2.09 × 10^-5^
Neurology	35.78 (17.50)	35.33 (18.72)	0.80
Orthopedic Surgery	63.72 (44.51)	47.92 (28.54)	1.75 × 10^-4^
Otolaryngology	80.38 (34.99)	49.92 (25.82)	1.83 × 10^-11^
Pathology	25.01 (14.90)	26.75 (11.65)	0.18
Pediatrics	29.56 (15.31)	29.11 (13.05)	0.99
Urology	79.01 (36.10)	49.78 (28.02)	3.02 × 10^-9^
Dermatology	83.84559 (±46.62186)	45.4031 (±33.38355)	5.50 × 10^-11^

**Figure 1:**
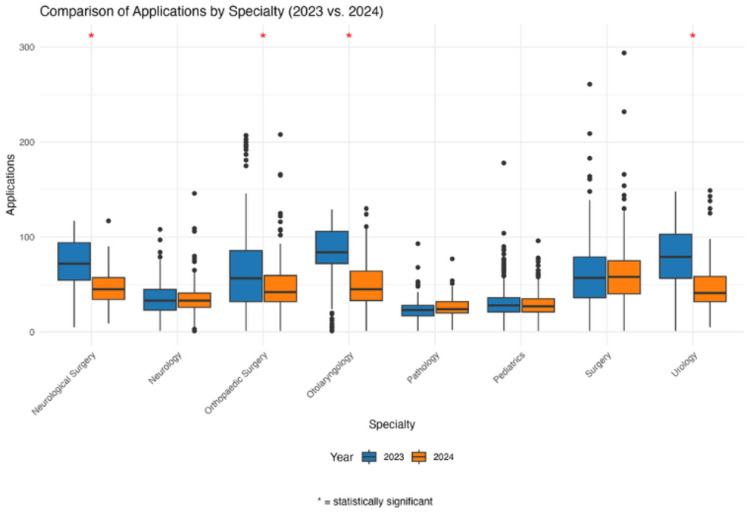
Number of Applications Submitted Across Eight Specialties Between 2023 and 2024 Box-and-whisker plot comparing the first application cycle in which Step 1 became pass/fail (2024). Red star above a specialty indicates a p-value < 0.05 in the Mann-Whitney U test.

However, other specialties, like general surgery (60.57 ± 37.01 to 59.44 ± 35.70), neurology (35.78 ± 17.49 to 35.33 ± 18.72), pathology (25.01 ± 14.90 to 26.75 ± 11.65), and pediatrics (29.56 ± 15.31 to 29.11 ± 13.05), showed no significant changes between the two years. These findings are visualized in Figure 1.

Average number of received interviews

The number of interviews remained relatively stable across most specialties. Only orthopedic surgery (12.78 ± 6.60 to 11.35 ± 5.71, p < 0.05) and pediatrics (16.75 ± 7.28 to 17.96 ± 7.23, p < 0.01) showed statistically significant changes.

Average step 2 scores

Step 2 scores remained largely consistent across specialties from 2023 to 2024, with the exception of pediatrics, which showed a statistically significant increase from 247.24 ± 13.52 to 248.84 ± 13.46 (p < 0.05).

**Figure 2:**
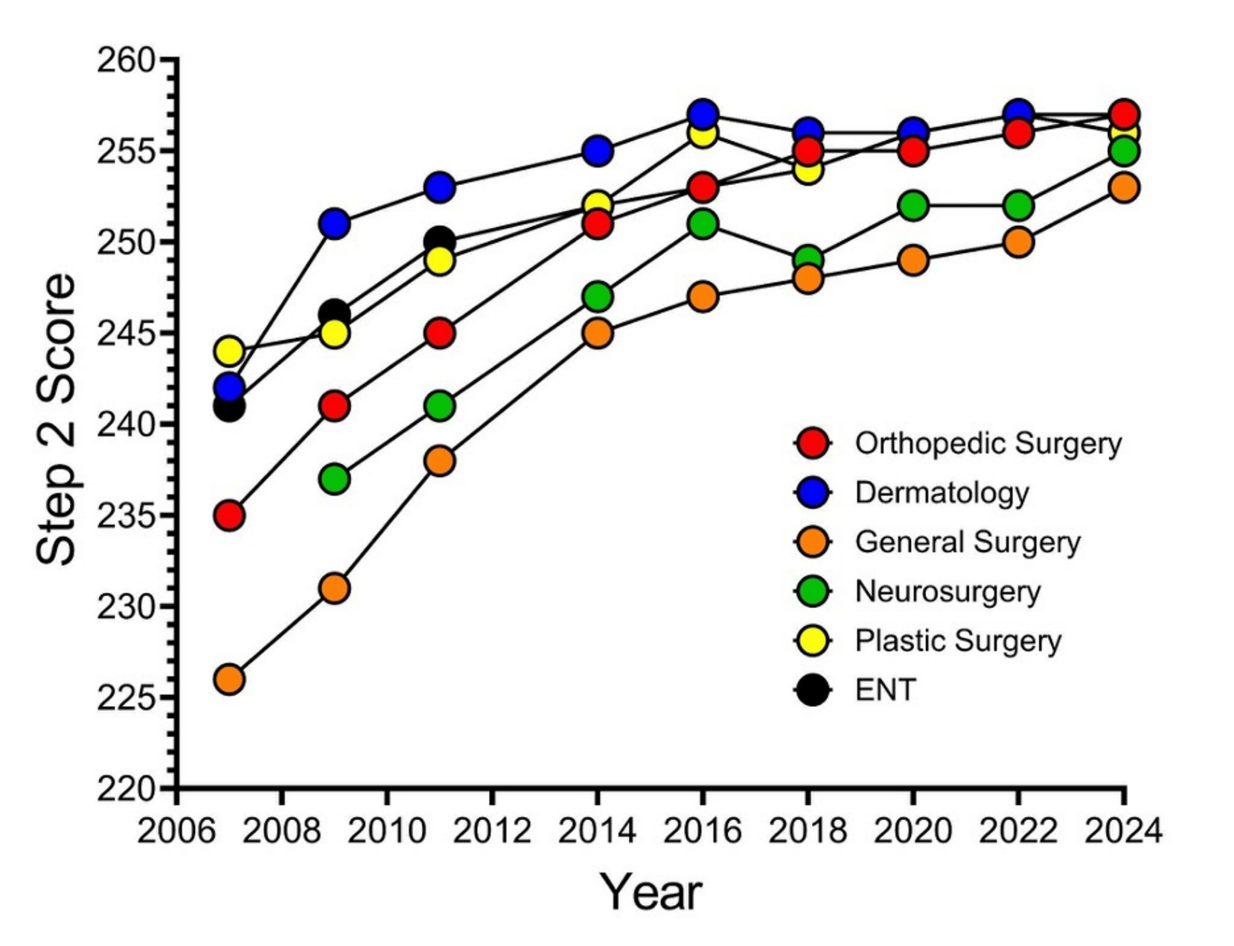
Matched Applicants’ Mean Step 2 Scores From NRMP’s Charting Outcomes in the Match Reports NRMP, National Resident Matching Program

Figure 2 shows a line graph depicting matched applicants’ mean Step 2 scores from NRMP’s Charting Outcomes in the Match reports.

Average number of research items

The number of research items did not show statistically significant changes for any specialty between 2023 and 2024. Although some specialties, like neurological surgery (10.76 ± 2.48 to 9.98 ± 3.18) and otolaryngology (9.59 ± 3.39 to 9.59 ± 3.36), maintained high numbers, and others, like general surgery (5.66 ± 3.99 to 6.23 ± 4.03) and pediatrics (3.88 ± 3.37 to 4.12 ± 3.54), showed slight increases, none of these changes were statistically significant.

**Figure 3:**
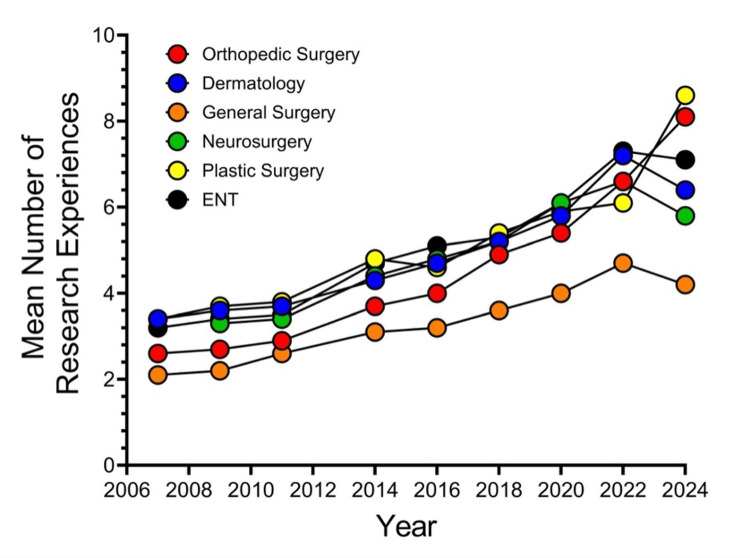
Matched Applicants’ Mean Number of Research Abstracts, Presentations, and Posters From NRMP’s Charting Outcomes in the Match Reports NRMP, National Resident Matching Program

Figure 3 shows a line graph depicting matched applicants’ mean number of research abstracts, presentations, and posters from the NRMP’s Charting Outcomes in the Match reports.

**Figure 4:**
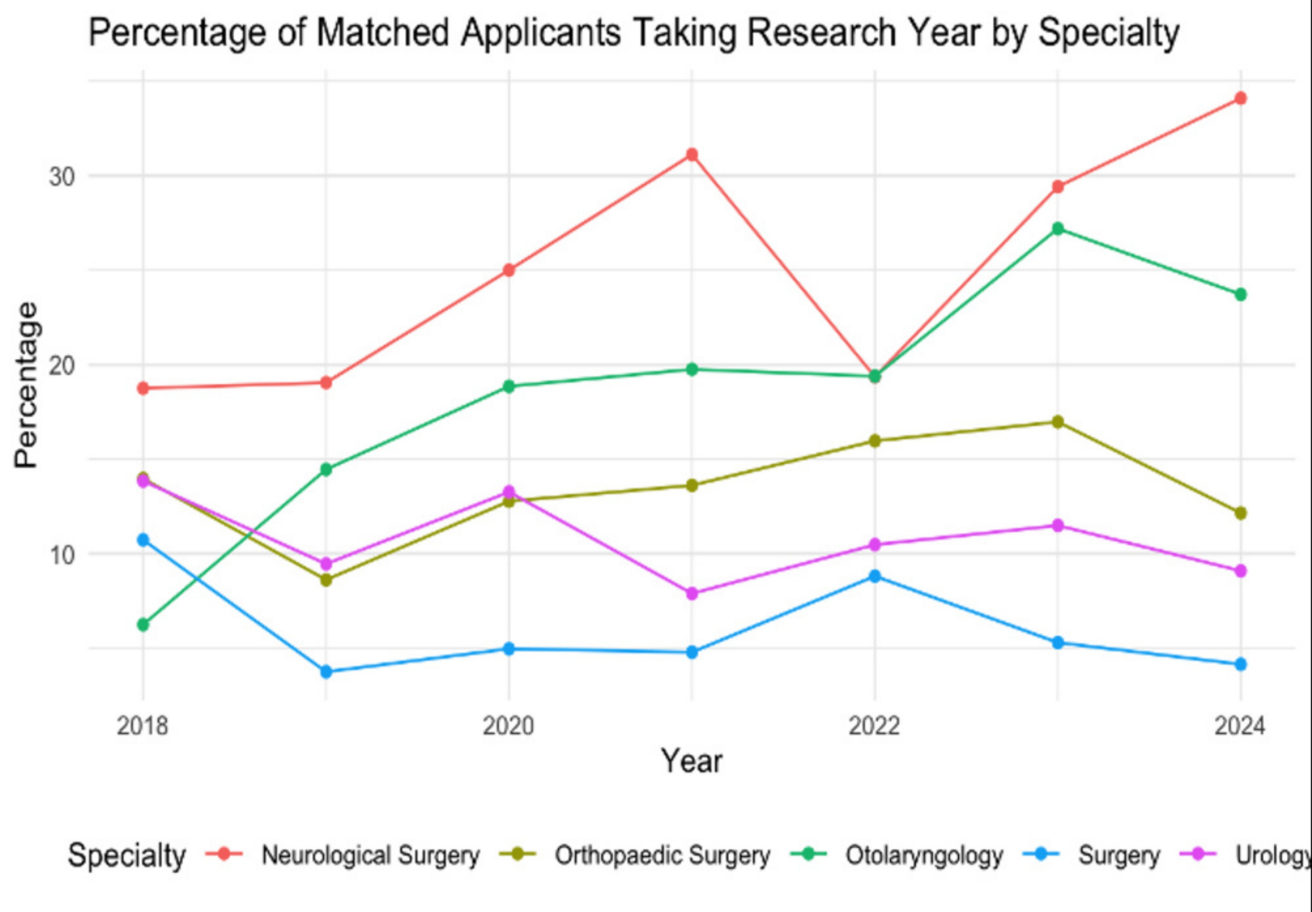
Percentage of Matched Applicants Taking Research Year by Specialty

Figure 4 reflects how the percentage of applicants taking a research year, for each specialty, has changed over time.

Average number of peer-reviewed publications

Publication numbers showed significant changes in some surgical specialties. Neurological Surgery saw a decrease from 9.02 ± 3.70 to 6.55 ± 4.47 (p < 0.01), along with Orthopedic Surgery (from 5.66 ± 4.33 to 4.79 ± 4.22; p < 0.05), Otolaryngology (from 6.92 ± 3.91 to 4.49 ± 3.63; p < 0.001) and Urology (from 5.28 ± 3.90 to 4.23 ± 3.70; p < 0.05). Other specialties did not show statistically significant changes in publication numbers.

Average number of volunteer experiences

Volunteer experiences showed significant decreases across all specialties. General surgery (7.93 ± 3.30 to 4.21 ± 2.44, p < 0.01), neurological surgery (7.10 ± 3.38 to 3.93 ± 2.78, p < 0.01), orthopedic surgery (7.87 ± 3.26 to 4.25 ± 2.44, p < 0.01), otolaryngology (7.90 ± 3.16 to 4.00 ± 2.59, p < 0.01), urology (6.06 ± 3.34 to 3.84 ± 2.42, p < 0.01), neurology (7.22 ± 3.25 to 4.08 ± 2.53, p < 0.01), pediatrics (8.34 ± 2.99 to 5.01 ± 2.85, p < 0.01), and pathology (5.30 ± 3.15 to 3.25 ± 2.63, p < 0.01) all experienced significant decreases in volunteer experiences from 2023 to 2024.

**Figure 5:**
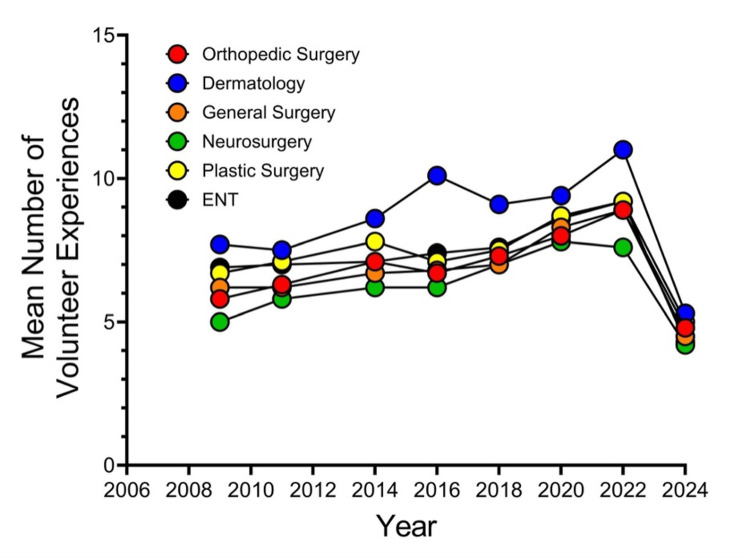
Matched Applicants’ Mean Number of Volunteer Experiences From NRMP’s Charting Outcomes in the Match Reports NRMP, National Resident Matching Program

Figure 5 shows a line graph depicting matched applicants’ mean number of volunteer experiences from NRMP’s Charting Outcomes in the Match reports.

Average number of leadership positions

General surgery saw a reduction from 5.00 ± 3.15 to 3.56 ± 2.27 (p < 0.01), along with neurological surgery (from 5.35 ± 2.81 to 3.80 ± 1.96; p < 0.01), orthopedic surgery (from 4.98 ± 2.91 to 3.69 ± 2.20; p < 0.01), otolaryngology (from 5.69 ± 3.12 to 3.93 ± 2.10; p < 0.01), and pediatrics (from 4.65 ± 2.78 to 3.32 ± 1.85; p < 0.01). Neurology (from 3.62 ± 2.56 to 3.64 ± 2.32, p > 0.05), pathology (from 2.91 ± 2.67 to 2.89 ± 2.62, p > 0.05), and urology (p > 0.05) did not show statistically significant changes.

Average number of clerkship honors

Clerkship honors increased in specialties such as orthopedic surgery (from 3.95 ± 2.59 to 4.73 ± 2.16; p < 0.01), otolaryngology (from 3.75 ± 2.62 to 4.81 ± 2.12; p < 0.01), neurology (from 2.99 ± 2.52 to 3.48 ± 2.25; p < 0.05), and pediatrics (from 2.86 ± 2.46 to 3.44 ± 2.33; p < 0.01). Other specialties, including general surgery (3.38 ± 2.62 to 3.75 ± 2.42), neurological surgery (4.02 ± 2.72 to 4.89 ± 2.09), pathology (2.41 ± 2.39 to 2.96 ± 2.24), and urology (3.54 ± 2.54 to 4.25 ± 2.31), did not show statistically significant changes in clerkship honors. These results are further reflected in Table 4.

**Table 4:** Changes in Number of Applications and Clerkship Honors Between 2023 and 2024

Specialty	2023 Clerkship Honors Mean (SD)	2024 Clerkship Honors Mean (SD)	Mann-Whitney U Test p-value
				General Surgery	3.38 (2.62)	3.75 (2.42)	0.066
Neurological Surgery	4.02 (2.72)	4.89 (2.09)	0.17
Neurology	2.99 (2.52)	3.48 (2.25)	0.043
Orthopedic Surgery	3.95 (2.60)	4.73 (2.16)	0.0037
Otolaryngology	3.75 (2.62)	4.81 (2.12)	0.0047
Pathology	2.41 (2.39)	2.96 (2.24)	0.091
Pediatrics	2.86 (2.46)	3.44 (2.33)	3.21 × 10^-5^
Urology	3.54 (2.54)	4.25 (2.31)	0.083

**Figure 6:**
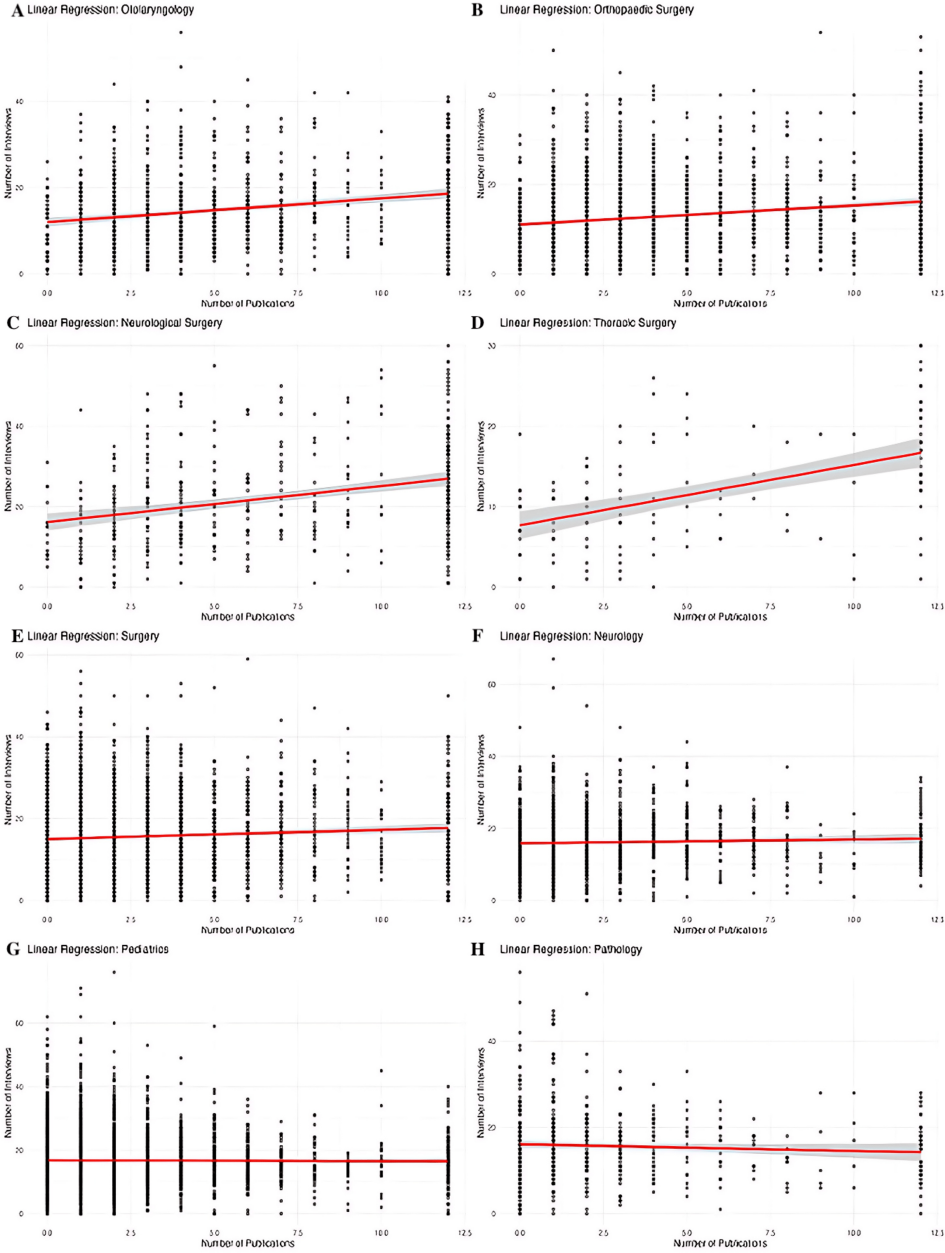
Linear Regression Model for the Number of Publications and Number of Interviews by Specialty Linear regression analyses demonstrate the association between the number of publications and the number of residency interviews for eight medical and surgical specialties. Each panel shows individual applicants as scatter points, with a fitted linear regression line (red) and a 95% confidence interval (gray). (A) Otolaryngology, (B) Orthopedic Surgery, (C) Neurological Surgery, (D) Thoracic Surgery, (E) General Surgery, (F) Neurology, (G) Pediatrics, (H) Pathology

**Figure 7:**
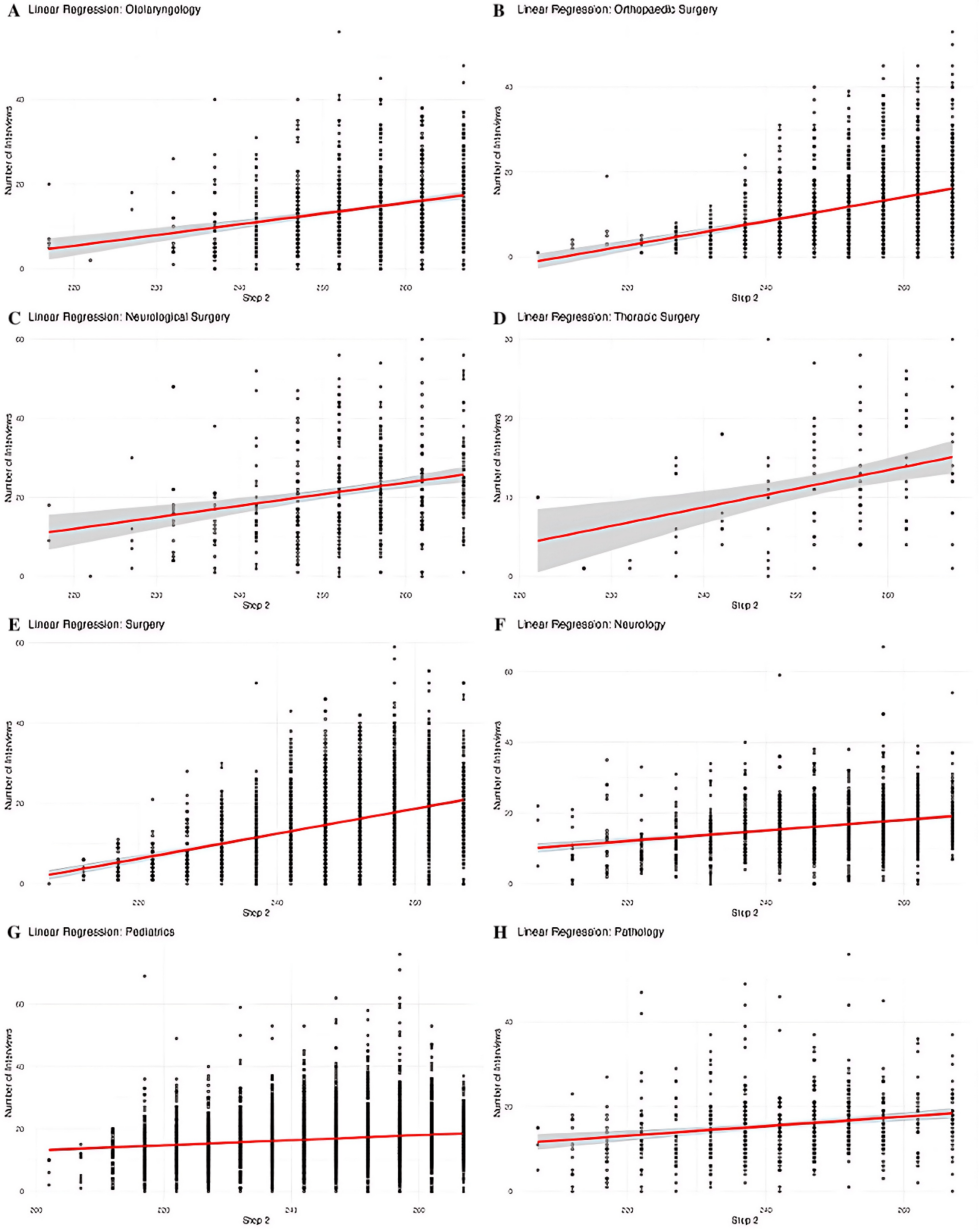
Linear Regression Model for Step 2 Scores and Number of Interviews by Specialty Linear regression analyses showing the association between United States Medical Licensing Examination (USMLE) Step 2 Clinical Knowledge (CK) scores and the number of residency interviews across eight specialties. Each scatterplot displays individual applicants, with a fitted linear regression line (red) and 95% confidence interval (gray). (A) Otolaryngology, (B) Orthopedic Surgery, (C) Neurological Surgery, (D) Thoracic Surgery, (E) General Surgery, (F) Neurology, (G) Pediatrics, (H) Pathology

Figure 6 shows a linear regression model for the impact of the number of publications on the number of interviews received for each specialty. Figure 7 shows a linear regression model for the impact of Step 2 scores on the number of interviews received.

General surgery

Clerkship honors were the strongest statistically significant predictor of receiving interviews among general surgery applicants, followed by Step 2 scores (estimate = 0.27, p < 0.001) and volunteer experiences (estimate = 0.26, p < 0.001).

Factors that showed a non-significant impact included leadership positions, abstracts, and publications.

Neurological surgery

The strongest statistically significant positive predictor for an interview in neurological surgery was publications (estimate = 0.98, p < 0.001), followed by clerkship honors (estimate = 0.60, p < 0.05) and Step 2 scores (estimate = 0.25, p < 0.001).

Factors that had a nonsignificant impact include abstracts, posters and presentations, volunteer experiences, and leadership positions.

Orthopedic surgery

The strongest significant positive predictor for an interview in orthopedic surgery was the number of clerkship honors (estimate = 0.66, p < 0.001). Other statistically significant positive predictors included Step 2 scores (estimate = 0.24, p < 0.001), publications (estimate = 0.35, p < 0.001), volunteer experiences (estimate = 0.29, p < 0.001), and leadership positions (estimate = 0.24, p < 0.01).

Abstracts, posters, and presentations had no significant impact (estimate = -0.014, p = 0.83) in predicting interviews received for orthopedic surgery. This falls in line with several factors that predict interviews in otolaryngology, except that there also appears to be greater emphasis on leadership positions in orthopedic surgery.

Otolaryngology

The biggest predictors for an interview in otolaryngology were the number of clerkship honors (estimate = 0.729687, p < 0.001), followed by publications (estimate = 0.53, p < 0.001). Other, more moderate positive predictors included volunteer experiences (estimate = 0.21, p = 0.04751) and Step 2 scores (estimate = 0.20, p < 0.001).

Factors that had a non-statistically significant impact on predicting an interview included abstracts, posters, and presentations (estimate = 0.09, p = 0.33), as well as leadership positions (estimate = 0.08, p = 0.5).

Urology

The strongest significant positive predictor for urology outcomes was the number of clerkship honors (estimate = 1.06775, p < 0.001). Other statistically significant positive predictors included research items (estimate = 0.43, p < 0.001), leadership positions (estimate = 0.36, p < 0.05), Step 2 scores (estimate = 0.11, p < 0.005), and number of applications (estimate = 0.03, p < 0.05).

Research years, publications, and volunteer experiences did not show statistically significant associations.

Neurology

The strongest predictors for receiving an interview in neurology included clerkship honors (estimate = 0.38, p < 0.001), followed by leadership positions (estimate = 0.32, p < 0.005), volunteer experiences (estimate = 0.25, p < 0.005) and Step 2 scores (estimate = 0.13, p < 0.001).

Pathology

For pathology, Step 2 scores had a statistically significant positive impact (estimate = 0.12, p < 0.001). However, other factors show no statistically significant effects in this specialty.

Pediatrics

For pediatrics, clerkship honors had the strongest impact (estimate = 0.36, p < 0.001), followed by volunteer experiences (estimate = 0.27, p < 0.001), leadership positions (estimate = 0.15, p < 0.05), abstracts, posters and presentations (estimate = 0.14, p < 0.05), and Step 2 scores (estimate = 0.05, p < 0.001).

Logistic regression model for predicting match status in surgical specialties

The logistic regression model for predicting all surgical residency match success identified three significant predictors: interviews (β = 0.11, p < 0.01), clerkship honors (β = 0.05, p < 0.01), and Step 2 scores (β = 0.01, p < 0.01). Among these, the number of interviews demonstrated the strongest positive effect on application success, followed by clerkship honors and Step 2 scores. The model's goodness of fit is reflected by a reduction in deviance from 4908.8 (null) to 4348.0 (residual), with an AIC of 4356. ROC curve analysis indicates moderate discriminative ability, with an AUC of 0.754, suggesting the model correctly classifies outcomes approximately 75.4% of the time.

## Discussion

General trends in various match factors

The transition of the USMLE Step 1 exam to P/F has significantly reshaped the residency application landscape, particularly within various competitive surgical specialties, such as otolaryngology, neurological surgery, orthopedic surgery, and urology. Our study reveals a marked shift in the importance of certain application metrics, including Step 2 scores and research productivity, suggesting that these factors may increasingly serve as primary indicators of applicant competitiveness. In analyzing trends from 2018 to 2024 across surgical and nonsurgical specialties, and in comparing 2023 application metrics to 2024, we observed a heightened emphasis on quantifiable academic and research achievements with the Step 1 scoring change, while experiential factors, such as leadership positions, have generally declined.

Step 2 scores and clerkship honors

We observed an increase in Step 2 scores for pediatrics, while no significant change was seen in other specialties. Across all specialties, the Step 2 score was consistently utilized as a moderate predictor for interviews, underscoring its overall importance in residency applications.

Across surgical and nonsurgical specialties, clerkship honors remain crucial in predicting interview invitations for applicants. For all specialties except pathology, clerkship honors were either the greatest or second-greatest predictor of interviews. This consistent finding emphasizes the value placed on clinical performance, professionalism, and evaluative feedback during rotations - all of which are components assessed through clerkship grading. Clerkship performance may offer a more holistic measure of readiness than standardized testing alone, as students are evaluated in real patient-care settings.

Despite their perceived utility, clerkship honors are not without controversy. As reliance on clinical evaluations grows, serious concerns have emerged about the objectivity and equity of clerkship grading. Previous work has shown that grading systems across institutions are inconsistent, often lacking transparent criteria, standardization, and structured training for evaluators - factors that can introduce bias and disproportionately affect students from underrepresented or non-traditional backgrounds [6]. Reform measures, such as grading committees, explicit rubrics, and improved transparency in Medical Student Performance Evaluation (MSPE) reporting, have been proposed to mitigate inequities and improve fairness in the residency selection process [6].

Moreover, while clerkship honors may influence interview offers, their ability to predict actual residency performance appears limited. A study of 258 general surgery graduates found only weak correlations between clerkship honors and performance ratings in domains such as surgical judgment, leadership, and medical knowledge [6]. Although honors in specific clerkships (e.g., surgery, OB/GYN, pediatrics), and a higher proportion of honors overall, were modestly associated with better evaluations, none of the measured application variables - including clerkship grades - were strong predictors of future success in residency [7]. These findings challenge the prevailing assumption that clerkship honors reliably identify top-performing residents and underscore the need to balance their use with more holistic, equitable, and longitudinally validated assessment methods [7].

The importance of clerkship honors across nearly all specialties, excluding pathology, may reflect their unique ability to capture interpersonal and hands-on competencies that standardized exams cannot measure. In surgical fields, especially, clerkship grades likely serve as a proxy for qualities such as adaptability, teamwork, and real-time clinical decision-making. By contrast, pathology programs may weigh clinical performance less heavily, due to the field’s more academic and analytical focus.

Research publications

Additionally, in our study, the number of publications was a primary or secondary predictor of interview offers for all surgical specialties, except urology and general surgery. The emphasis on research publications not only reflects academic rigor but also signals a candidate’s engagement with the specialty and potential for future academic contributions. This could be due, in part, to how several of these specialties have a strong academic presence and many programs based in research-intensive institutions, which may lead students to feel increased pressure to enhance their research efforts [8]. With fewer objective standardized metrics available currently, the weight placed on research may continue to rise, as program directors seek additional data points to differentiate between otherwise similar applicants.

Decrease in volunteer and leadership experiences

The statistically significant decrease in volunteer and leadership experiences across both surgical and nonsurgical specialties may suggest a reallocation of time from these pursuits toward research and academics. This trend may also be due to the constraints placed on applicants by the Electronic Residency Application Service (ERAS), an online platform used by graduating medical students to apply to residency programs. In 2024, the Association of American Medical Colleges (AAMC) changed the ERAS application to allow only up to 10 experiences, including work, teaching/mentoring, education/training, research, and leadership positions in extracurricular activities, as well as volunteer experiences. This recent change has also contributed to the decline of volunteer and leadership experiences, as this limit now encourages applicants to recount their most meaningful engagements rather than compiling an exhaustive list of achievements [9].

More importantly, emerging research supports this shift in emphasis. One review found no significant correlation between the number of volunteer hours in student-run free clinics and improved residency match outcomes [9]. Instead, the study emphasized that longitudinal, meaningful involvement - particularly in roles reflecting commitment and impact - was more predictive of applicant competitiveness. These findings suggest that depth of experience, rather than high-volume volunteering, holds greater value in signaling a candidate’s dedication to service and overall readiness for residency [10]. This may help explain why residency programs have placed less emphasis on the sheer number of volunteer and leadership experiences and, instead, place greater value on sustained involvement, prompting applicants to prioritize their engagement and invest more deeply in fewer, more impactful roles.

It is worth noting that, despite the general decline in volunteer and leadership experiences across applicants, these factors remain moderately positive predictors for interviews in pediatrics, neurology, orthopedic surgery, and otolaryngology. However, this general trend raises important questions regarding the interpersonal competencies of applicants, which are also vital for patient care. Given this variability, it is necessary for programs to carefully reflect on how they weigh different experiences in their selection criteria.

Limitations

Due to the nature of data collection for the Texas STAR database, the main limitation of this study is the potential for self-selection bias in the optional survey. It is important to acknowledge that the Texas STAR dataset primarily comprises applicants who successfully matched. As a result, the predictive strength of certain metrics may be overestimated, and the findings may not fully reflect the characteristics of unmatched applicants. Furthermore, the Texas STAR database does not capture qualitative factors such as letters of recommendation, personal statements, and interview performance, all of which play influential roles in residency selection and limit our findings. Additionally, variability in medical school curricula, institutional differences in grading systems, access to research opportunities, and advising practices create significant constraints on the conclusions that can be drawn from the data, as different medical schools may have differing timelines and requirements. Since not all U.S. medical schools participate in the Texas STAR database, the generalizability of the findings at a national level may also be limited. The rapidly evolving residency application environment, particularly surrounding the Step 1 P/F transition, may limit the replicability of our findings in future cycles. Lastly, exclusively using quantitative data prevents a qualitative understanding of applicants’ motivations throughout the application process.

## Conclusions

In line with program directors' predictions, applicants appear to be focusing more heavily on Step 2 scores as a differentiating factor since the change of Step 1 to P/F. While Step 2 scores for surgical specialties remained stable between 2023 and 2024, we observed significant increases in non-surgical specialties, suggesting that applicants in fields without intensive technical skills requirements may place even greater emphasis on this metric. The increase in abstracts, presentations, and publications across many specialties underscores the importance of research productivity as a core factor. Given the rising number of applicants engaging in research, these findings support the notion that scholarly output is now a more critical component of applications, likely due to its ability to demonstrate academic rigor and a commitment to specialty-specific inquiry.

For both applicants and residency program directors, these changes can provide insight into choosing applicants and enhancing their applications. From the perspective of residency program directors, we see a shift in application metrics - including greater importance of research productivity and clerkship honors, along with a deemphasis on leadership and volunteer activities - that can allow them to reflect on their current criteria for selection of future surgical and nonsurgical specialty applicants. This, in turn, can help them determine how they weigh research and academic success compared with leadership and volunteer experiences and, thus, the types of applicants they hope to attract and who they believe will have the most success in the field during future practice. These changes also highlight the need for medical schools to adopt advising strategies to assist applicants in planning their research projects and academic preparedness for Step 2 and clerkships. Such support is essential to fostering candidates who can achieve success in matching into competitive surgical specialties such as otolaryngology, neurological surgery, orthopedic surgery, and urology.
